# Hypoxia-induced the upregulation of stromal cell-derived factor 1 in fibroblast-like synoviocytes contributes to migration of monocytes into synovium tissue in rheumatoid arthritis

**DOI:** 10.1186/s13578-018-0210-x

**Published:** 2018-02-14

**Authors:** Ru Yang, Yanhua Yao, Panjun Wang

**Affiliations:** 1 Department of Rheumatology, The Second Affiliated Hospital of Soochow University, Soochow, Jiangsu China; 2 Department of Hematology and Oncology, The Children’s Hospital of Soochow University, Soochow, Jiangsu China; 3 Department of Hematology, The Second Affiliated Hospital of Soochow University, Soochow, Jiangsu China

**Keywords:** Rheumatoid arthritis, Hypoxia, Fibroblast-like synoviocytes, Monocytes, SDF-1, CXCR4, Inflammatory factors

## Abstract

**Background:**

Rheumatoid arthritis (RA) is an auto-immune disease characterized by chronic inflammation of multiple joints. Hypoxia is a constant feature of synovial microenvironment in RA. Fibroblast-like synoviocytes (FLSs), which are potent effector cells in RA. It has been reported that large numbers of monocytes are recruited to the synovium and play an important role in synovial inflammation and tissue destruction in RA. However, the mechanism is still unclear. The aim of this study is to explore the role of hypoxia microenvironment on the recruitment of monocytes and then promote the development of RA.

**Methods:**

Rheumatoid arthritis model was constructed. Monocytes and FLSs were isolated from rheumatoid arthritis mice. RT-PCR, western blot and ELISA were used to detect the expression of SDF-1 in FLSs. CXCR4 expression in monocytes was examined by cell immunofluorescence and flow cytometry analysis. Transwell assay was performed to evaluate the potential of cell migration.

**Results:**

We demonstrated that hypoxia microenvironment enhanced SDF-1 production of FLSs, which attracted the recruitment of CXCR4-expressing monocytes to the synovium and induced monocytes differentiation into tissue macrophages. Moreover, these macrophages secreted inflammatory factors including IL-6, TNF-α, IL-1β and MMP-3, which contributed to the synovial inflammation and tissue destruction in RA.

**Conclusion:**

The results of this study suggested that hypoxia microenvironment played an important role in enhancing SDF-1 production of FLSs. SDF-1/CXCR4 axis was involved in the recruitment of monocytes in RA synovium and it might be a possible way of inhibiting inflammation and bone erosion in RA.

## Background

Rheumatoid arthritis (RA) is an auto-immune disease characterized by chronic inflammation of multiple joints. RA leads to the synovial membrane of diarthrodial joints and damages the articular tissues, resulting in severe functional disarrangement of the joints. It affects 1% of the world population and treatment has been revolutionized by the use of biologic therapies, such as drugs that target cytokines, cells and signaling pathways [1]. Fibroblast-like synoviocytes (FLSs), which are potent effector cells in RA, generating enzymes that degrade cartilage and bone and serving as a primary source of inflammatory cytokines in the synovium [2–4].

There is an influx of immune cells in RA, which causes and maintains inflammation [5]. These immune cells consist mainly of T lymphocytes and macrophages/monocytes, which together with the resident cell types, such as FLSs, contribute to the disease pathology [6–8]. Monocytes contribute to RA pathogenesis in part by producing inflammatory cytokines and chemokines [9]. It has been shown that large numbers of monocytes are recruited to the synovium and monocytes play an important role in synovial inflammation and tissue destruction in RA [10–12]. Rheumatoid FLSs display features of “nurse-like” stromal cells in their ability to support the spontaneous migration of immune cells [13]. Targeting monocytes/macrophages should be a possible way of inhibiting inflammation and bone erosion in arthritis. Therefore, understanding the key factors in synovial microenvironment that modulate monocytes infiltration in synovium is important for the therapy of RA.

Hypoxia is a constant feature of synovial microenvironment in RA. Synovial hypoxia is thus considered a potential pathogenic factor in RA. It has been demonstrated that oxygen tension in the synovial fluid of RA patients was found to be lower than that in healthy controls and osteoarthritis patients [14, 15]. Our previous work also demonstrated that HIF-1α was significantly up-regulated in rheumatoid arthritis tissue [16]. Additionally, research has been suggested that hypoxia correlated with the intensity of the inflammatory process and cell migration in RA, which indicating hypoxia participated in the inflammatory process [17]. However, the mechanism between hypoxia and inflammation in RA is still unclear. Here, we investigated the role of hypoxia on regulating the “nurse-like” stromal cells (FLSs) and the mechanism of monocytes infiltration in synovium.

## Methods

### Specimens

Synovial tissues were obtained at surgery from 10 RA patients with the average age 51.4-year-old. RA was diagnosed according to the American College of Rheumatology criteria and these cases have the same degree of RA [18]. Informed consent was obtained from the patients and the protocol was approved by an ethical committee of the Second Affiliated Hospital of Soochow University. Any experimental research on humans was in compliance with the Helsinki Declaration.

### Collagen-induced arthritis in mice

All of Male DBA/1 mice were used at 6–8 week old. All of the procedures were approved and conducted according to the Institutional Animal Care and Committee Guide of Soochow University. To induce CIA in mice, 0.1 ml of an emulsion containing 100 μg of bovine type II collagen and an equal volume of Freund’s complete adjuvant (Arthrogen-CIA) were injected intradermally into the base of the tail as a primary immunization. 21 days later, 100 μg of type II collagen dissolved and emulsified 1:1 with Freund’s incomplete adjuvant (Difco) was administered to the hind leg as a booster injection. Mice were monitored for signs of arthritis three times a week by two blinded observers. The mice were sacrificed at days 38–40 and joint tissues were obtained, seven mice were used in each group.

### Isolation and culture of monocytes and fibroblast-like synoviocytes

Peripheral blood monocytes were isolated by density-gradient centrifugation using Ficoll-Hypaque (Pharmacia LKB) from mice. In order to isolate FLSs, after removal of the paw bones from mice, the joint cavity was cut open to reveal the synovial tissues, which were sliced into 1 mm^3^ pieces. These pieces were washed with Dulbecco’s modified Eagle’s medium (DMEM) (GIBCO, Invitrogen) with 10% fetal bovine serum (FBS) and transferred to a culture bottle for culture in DMEM containing 10% FBS, 100 IU/ml penicillin, and 100 μg/ml streptomycin at 37 °C in 5% CO_2_ for 24 h. Cells were passaged at a ratio of 1:2, and passages 3–5 of the cells were used. Normal FLSs were obtained from control mice, and RA-FLSs were obtained from CIA mice. FLSs were treated with 1% O_2_ considered as hypoxic condition.

### Immunohistofluorescence

Immunohistofluorescence assay was performed according to the method described before [19]. Anti-α-SMA antibody and anti-F4/80 (Abcam, UK) were used. All of these antibodies were used at a dilution of 1/100. Alexa 488-conjugated goat anti-mouse IgG (Invitrogen, Carlsbad, CA) and Alexa 568-conjugated goat anti-rabbit IgG (Invitrogen) were used as secondary antibodies.

### Cell immunofluorescence staining

Cells were fixed in 4% paraformaldehyde solution for 15 min and then were blocked with PBS buffer containing 1% BSA at 37 °C for 30 min after washing with PBS. CXCR4 (Abcam, UK) antibody was added to cells and incubated overnight at 4 °C. Alexa Fluor 488 goat anti-rabbit IgG (Invitrogen) secondary antibody was used at a dilution of 1:200. Nuclei were stained with DAPI (1 μg/ml, Sigma-Aldrich). Fluorescence intensity was evaluated using a confocal microscope (Olympus Corp., Japan).

### Flow cytometry analysis

2 × 10^5^ cells were used for each sample and then were labelled with respective antibodies anti-mouse-CD14-APC, anti-mouse CXCR4-FITC, anti-mouse CD36-FITC and anti-mouse F4/80-APC for 30 min in the dark and fixed in 1% paraformaldehyde and analysed by flow cytometry.

### Real-time quantitative PCR

Total RNA was isolated using TRIZOL (Invitrogen, Carls-bad, CA, USA) and cDNA synthesis was performed using the PrimeScript RT reagent Kit (Takara, Kyoto, Japan) according to the manufacturer’s instructions. The mRNA expression of SDF-1 and inflammatory factors (IL-6, TNF-α, IL-1β and MMP-3) were quantified by real-time quantitative PCR. Quantitative PCR was performed using SYBR Green PCR Kit (Applied BI) according to the manufacturer’s instructions.

### Enzyme-linked immunosorbent assay (ELISA)

To assess the secretion of SDF-1 and inflammatory factors, serum-free supernatant from FLSs and monocytes cultures after different treatment was collected and tested in triplicate by enzyme-linked immunosorbent assay (ELISA, R&D Systems) according to the manufacturer’s instruction.

### Migration assay

Cell migration was detected by the transwell assay. Hypoxia-disposed FLSs were added to the lower wells and mononuclear cells suspended in RPMI 1640 were added to the upper wells. After 24 h of incubation at 37 °C, the cells were fixed in 4% formaldehyde and stained with crystal violet dye and the cells that invaded through the pores to the lower surface of the filter were counted under a microscope. Three invasion chambers were used per condition. The values obtained were calculated by averaging the total number of cells from three filters. Additionally, to examine the role of SDF-1/CXCR4 axis in the migratory ability of monocytes, mononuclear cells were disposed with CXCR4 inhibitor (AMD3100, 1 μg/ml).

### Western blotting analysis

Western blot was performed according to a previous study [20]. Rabbit monoclonal anti-SDF-1, anti-pho-IκB antibody (1:1000, Abcam, UK), and a rabbit monoclonal anti-GAPDH antibody (1:5000; Bioworld Technology, St. Louis, MN, USA) were used. Western blot were repeated three times for each sample.

### Statistical analysis

All of the experiments were repeated at least three times. Analysis of variance was performed using GraphPad Prism 5.0 (GraphPad Software). Quantitative data were expressed as mean ± SD for each experiment. Significance between groups was performed by a Student’s test. P < 0.05 was considered statistically significant.

## Results

### Macrophages were adjacent to fibroblast-like synoviocytes in RA tissue

**Fig. 1:**
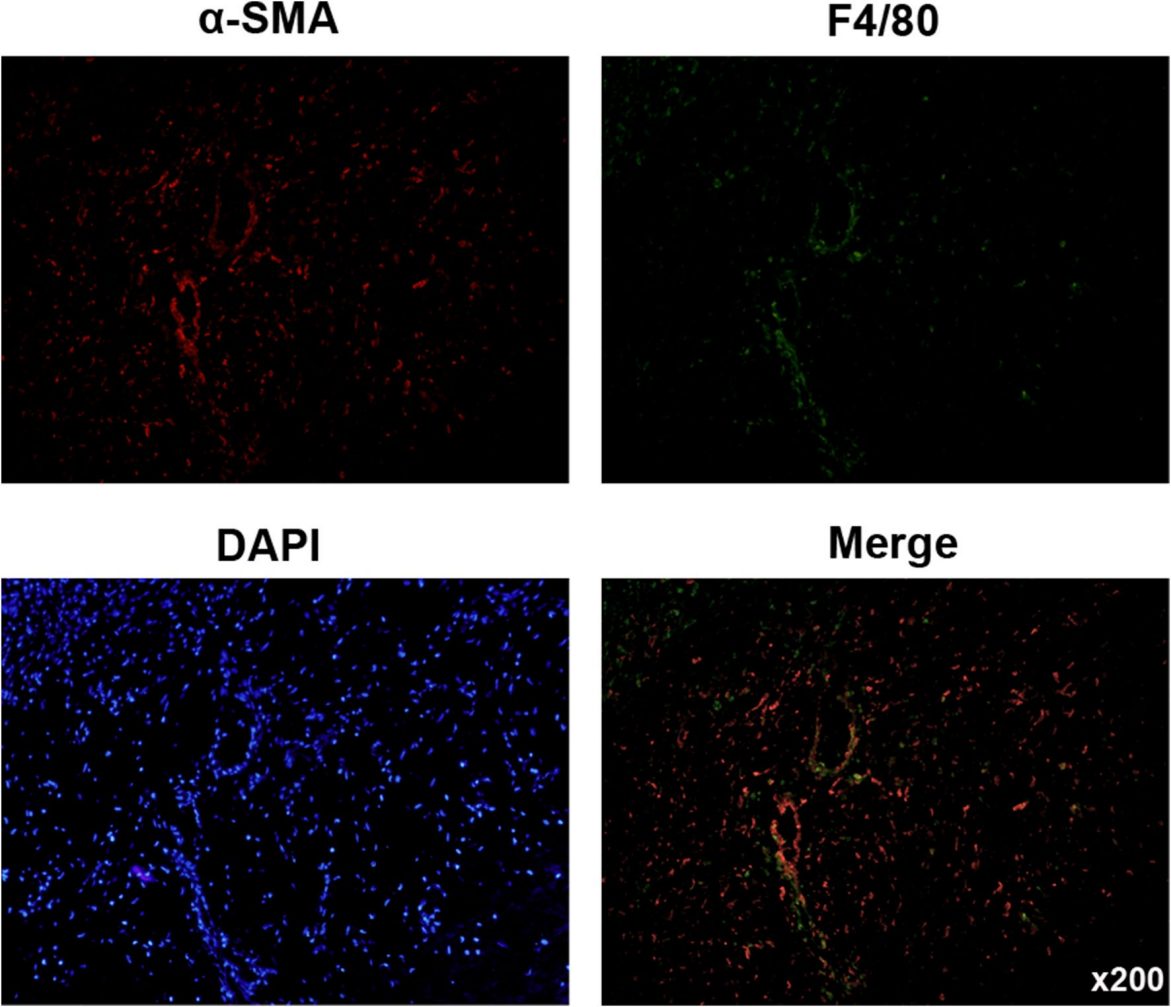
Macrophage was adjacent to fibroblast-like synoviocytes in RA tissue. Macrophage (F4/80, green) and fibroblast-like synoviocytes (α-SMA, red) expression was detected in RA tissue by immunohistofluorescence stain

Firstly, the relationship between macrophage and FLSs was analyzed in RA specimens. We detected F4/80 and α-SMA expression in synovium of RA by immunofluorescence. The results showed that F4/80-expressing macrophage was adjacent to α-SMA-expressing FLSs in RA synovium (Fig. 1), which indicated that monocytes were migrated to the synovium of RA.

### Monocytes expressed high level of CXCR4

**Fig. 2:**
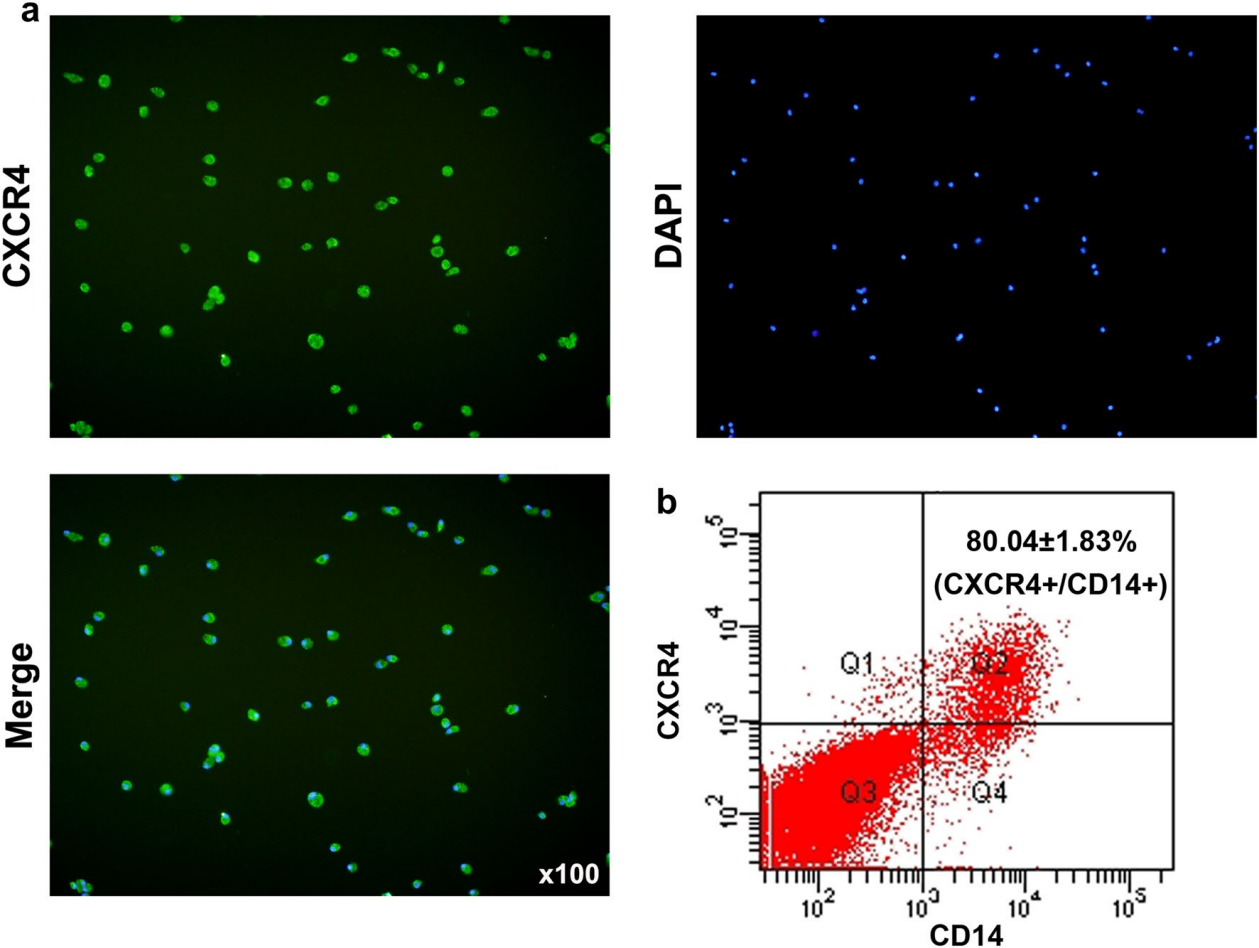
Monocytes expressed high level of CXCR4. **a** CXCR4 expression in monocytes was examined by cell immunofluorescence. **b** CD14 and CXCR4 expression in monocytes was examined by flow-cytometry analysis

To investigate the mechanism of monocytes migration to synovium, the key factor CXCR4 expression was examined by immunofluorescence and flow-cytometry analysis. We found that CXCR4 was expressed at a high level in monocytes by immunofluorescence detection (Fig. 2a). In consistent, there were 80.04 ± 1.83% CXCR4+ cells in CD14+ monocytes by flow-cytometry analysis (Fig. 2b). These results indicated that monocytes expressed high level of CXCR4.

### Hypoxia induced SDF-1 expression in fibroblast-like synoviocytes

**Fig. 3:**
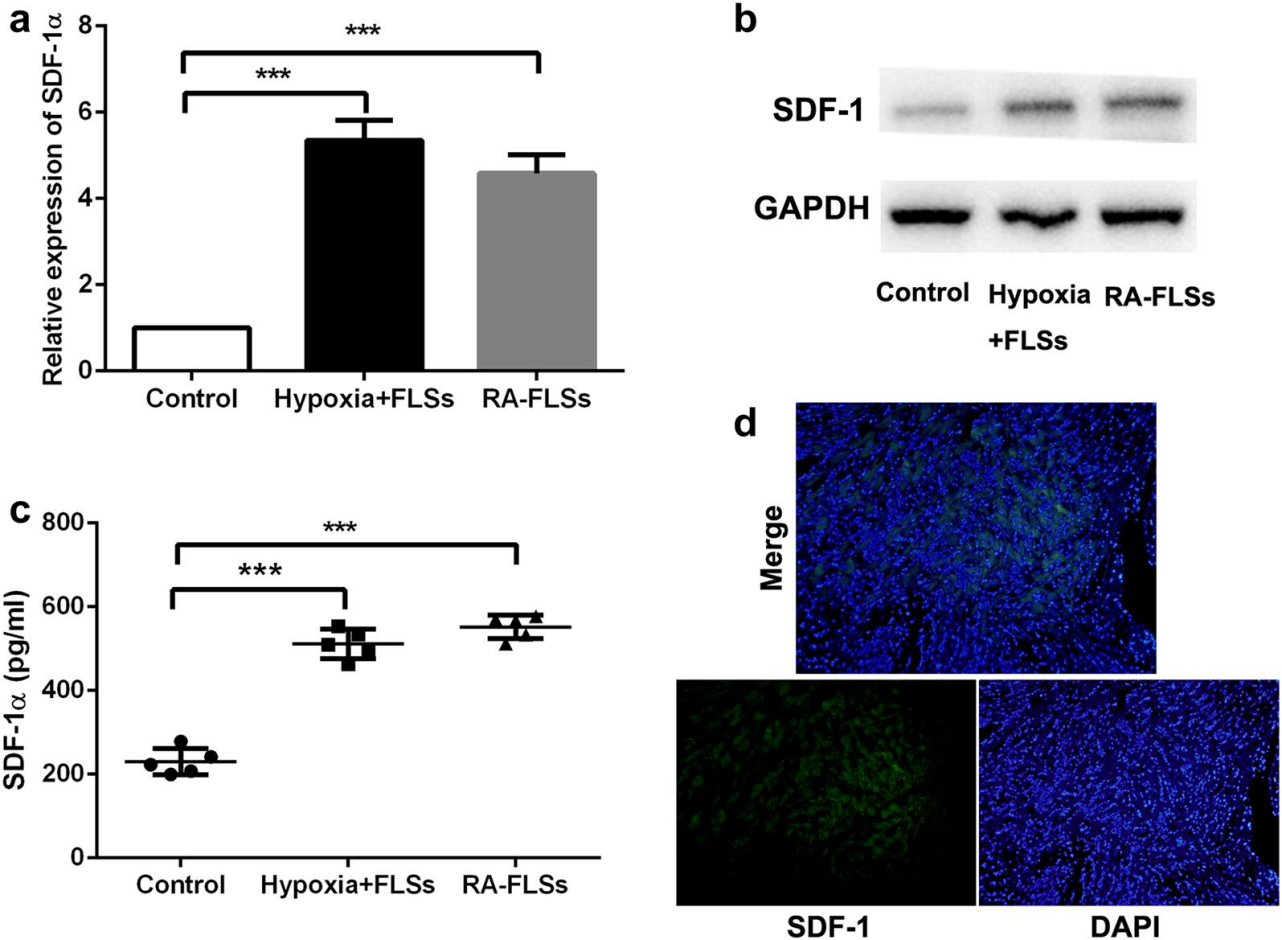
Hypoxia induced SDF-1 expression in fibroblast-like synoviocytes. **a** RT-PCR was performed to detect SDF-1 level in fibroblast-like synoviocytes under hypoxia stimulation. ***P < 0.001. **b** Western blot was performed to detect SDF-1 level in fibroblast-like synoviocytes under hypoxia stimulation. **c** ELISA was performed to detect SDF-1 level in fibroblast-like synoviocytes under hypoxia stimulation. ***P < 0.001. **d** SDF-1 level was investigated in RA synovial tissue by immunofluorescence

Our previous study indicated that hypoxia microenvironment was present in synovium of RA and FLSs were the potent effector cells [16]. FLSs derived from normal mice were disposed by hypoxia (1% O_2_) in vitro. In further, CXCR4 ligand, SDF-1 level was detected in FLSs, hypoxia-disposed FLSs and RA-FLSs by RT-PCR, western blot and ELISA. The results showed that the expression SDF-1 was upregulated in RA-FLSs and FLSs by hypoxia treatment (Fig. 3a–c). Additionally, SDF-1 level in RA specimens was also investigated and we found that SDF-1 was expressed at a high level in synovial tissue of RA cases (Fig. 3d). These results indicated that the hypoxia microenvironment that existed in RA tissues might play an important role in promoting the expression of SDF-1 in FLSs.

### Hypoxia induced SDF-1 expression through activation of NF-κB signaling pathway

**Fig. 4:**
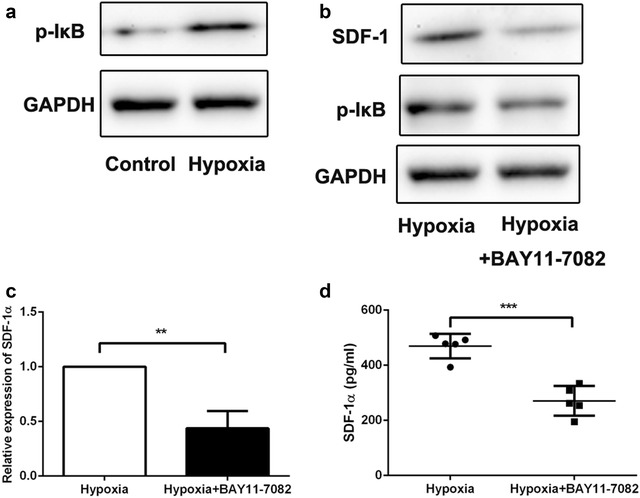
Hypoxia induced SDF-1 expression through activation of NF-κB signaling pathway in fibroblast-like synoviocytes. **a** Phosphorylate-IκB expression was detected by western blot in hypoxia-treated fibroblast-like synoviocytes. **b** Phosphorylate-IκB and SDF-1 expression was measured by western blot in hypoxia-treated fibroblast-like synoviocytes with dispose of NF-κB inhibitor, BAY 11-7082. **c** RT-PCR was performed to examine SDF-1 level in in hypoxia-treated fibroblast-like synoviocytes with dispose of NF-κB inhibitor, BAY 11-7082. **d** ELISA was performed to examine SDF-1 level in in hypoxia-treated fibroblast-like synoviocytes with dispose of NF-κB inhibitor, BAY 11-7082

Nuclear factor-κB (NF-κB) is a critical transcriptional activator of HIF-1α [21]. Therefore, we next examined whether hypoxic induction of SDF-1 occurred specifically through an NF-κB-dependent pathway. To investigate the activation of NF-κB pathway, western blot was used to detect the IκB activation. The results showed that phosphorylated-IκB was activated in FLSs with hypoxia treatment (Fig. 4a). In addition, BAY11-7082, a NF-κB inhibitor was used to block NF-κB signaling, then hypoxia-induced SDF-1 level was detected. The results demonstrated that SDF-1 level was attenuated when NF-κB signaling pathway was blocked (Fig. 4b–d). These findings indicated that hypoxia enhanced SDF-1 expression in FLSs by activating NF-κB signaling pathway.

### SDF-1/CXCR4 axis mediated the migration of monocytes

**Fig. 5:**
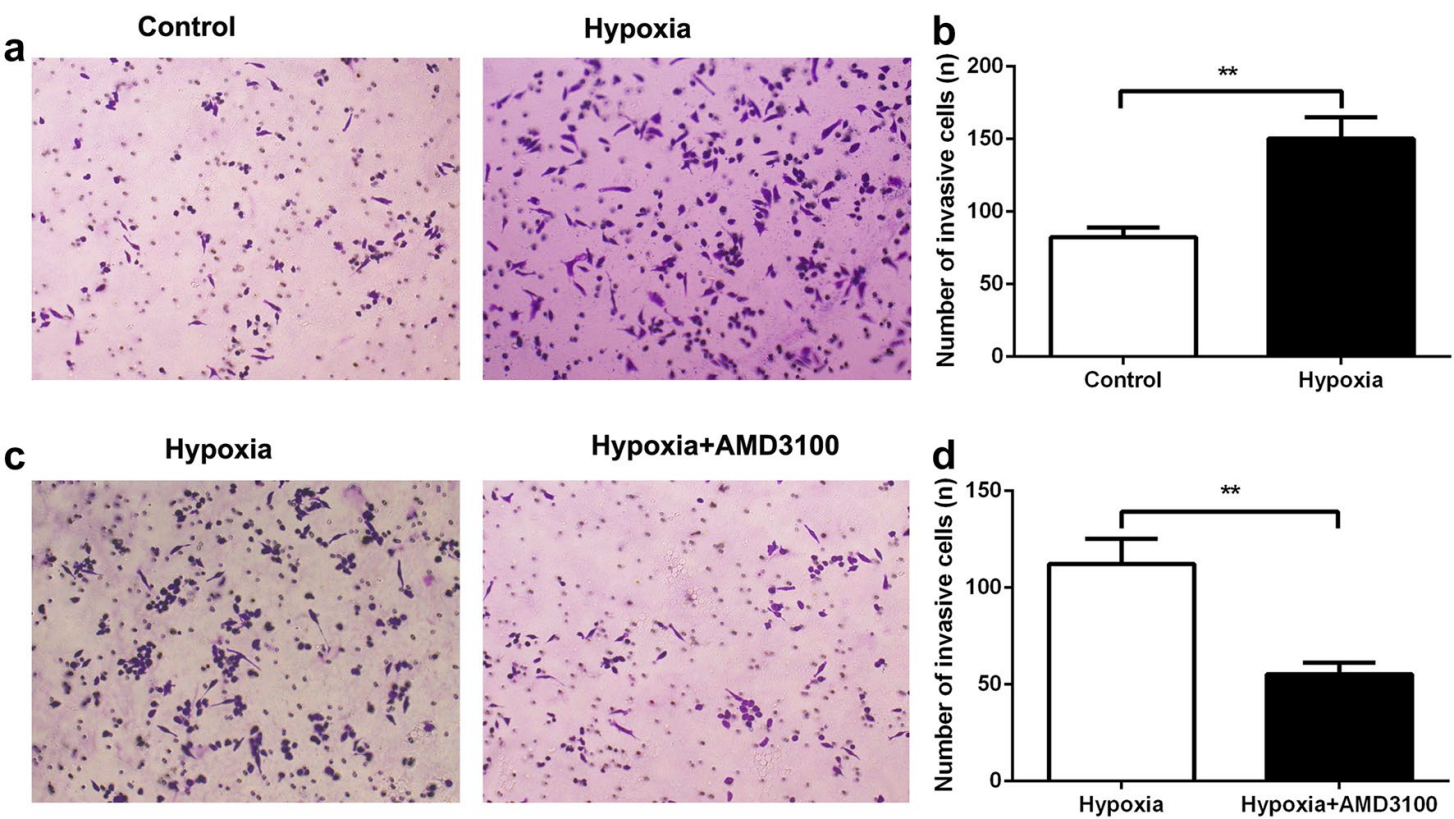
SDF-1/CXCR4 axis mediated the migration of monocytes. **a** and **b** Transwell assay was employed to detect the migratory ability of monocytes with the treatment of hypoxia-disposed FLSs’ cultured medium. **P < 0.01. **c** and **d** Transwell assay was performed to detect the migratory ability of monocytes with the treatment of hypoxia-disposed FLSs’ cultured medium; monocytes were conducted with CXCR4 inhibitor (AMD3100). **P < 0.01

Next, transwell assay was performed to examine the role of SDF-1/CXCR4 axis on the migration of monocytes. Monocytes were plated in the upper chamber and FLSs with or without hypoxia treatment were added in the lower chamber. We found that hypoxia-treated FLSs could significantly increase the migratory ability of monocytes (Fig. 5a, b), which indicated that high level of SDF-1 induced the invasive capacity of monocytes. In order to confirm the role of CXCR4 in the invasion, monocytes were disposed by a CXCR4 inhibitor, AMD3100. We found that the migratory ability was reversed when CXCR4 was inhibited in monocytes (Fig. 5c, d). These results indicated that SDF-1/CXCR4 axis played an important role in the monocytes infiltration in synovium and stroma-monocyte cell interactions.

### Hypoxia-treated fibroblast-like synoviocytes induced monocytes differentiated into macrophages and secreted inflammatory factors

**Fig. 6:**
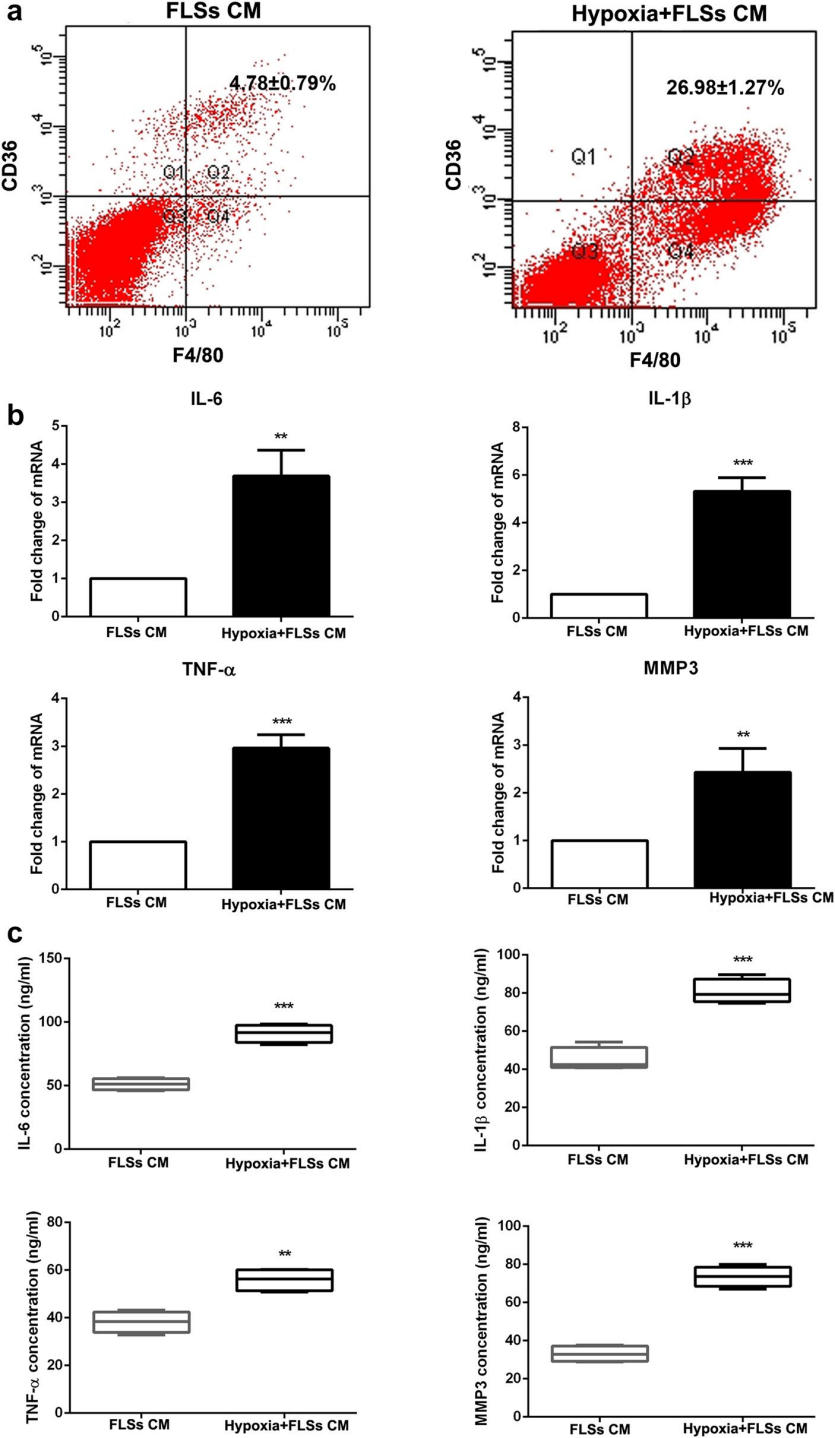
Hypoxia-treated fibroblast-like synoviocytes induced monocytes differentiated into macrophages and secreted inflammatory factors. **a** Expression of macrophages markers were measured by RT-PCR with the treatment of hypoxia-disposed FLSs’ culture medium. **b** and **c** Inflammatory factors (IL-6, TNF-α, IL-1β and MMP-3) levels were detected by RT PCR and ELISA in monocytes with the treatment of hypoxia-disposed FLSs’ culture medium. **P < 0.01, ***P < 0.001

To detect the interaction of RA-FLSs and monocytes, monocytes were treated with cultured medium obtained from hypoxia-disposed FLSs. As shown in Fig. 6a, hypoxia-disposed FLSs could induce monocytes differentiation into macrophages. Furthermore, these macrophages expressed a significant high level of inflammatory factors, such as IL-6, TNF-α, IL-1β and MMP-3 at mRNA and protein level (Fig. 6b, c). The data mentioned above indicated that monocytes differentiated into macrophages in synovial tissues under the microenvironment of RA and produced inflammatory factors, which lead to the synovial inflammation and tissue destruction in RA.

## Discussion

We previously reported that hypoxia microenvironment was existed in rheumatoid arthritis tissue. Now we found that hypoxia induced FLSs secrete high level of SDF-1 through NF-κB signaling pathway. It has been established that SDF-1 is one of the most efficacious chemoattractant for multiple leukocytes, including monocytes. The most common receptor of SDF-1, CXCR4, was expressed in monocytes. In our present work, we found that SDF-1/CXCR4 axis was involved in the migration of monocytes into synovial tissues. In the hypoxia microenvironment of rheumatoid arthritis tissue, monocytes secreted several inflammatory factors, such as IL-6, TNF-α, IL-1β and MMP-3, which contributed to the joint inflammation in RA.

The stromal cell-derived factor-1 (SDF-1) was expressed by most of RA synovial tissues. In addition, SDF-1 mRNA was expressed by cultured RA synoviocytes. Of note, previous studies indicated that cultured RA fibroblasts produce SDF-1 protein [22]. Additionally, it has been demonstrated that hypoxia stimulation markedly enhanced SDF-1 production [23]. In consistent with this, we found that hypoxia stimulation appeared to play an important role in the SDF-1 secretion of synovial fibroblasts in the rheumatoid synovium. Moreover, it is clear that NF-κB is a critical transcriptional activator of HIF-1α [21]. There are several studies demonstrating the cross-talk between the NF-κB and HIF-1 signaling pathways [21, 24–26]. To investigate this further, the role of NF-κB in hypoxia-induced SDF-1 production was detected in our study. The result showed that NF-κB signaling pathway was activated by hypoxia in RA-FLSs and SDF-1 expression was downregulated when NF-κB pathway was blocked in RA-FLSs with hypoxia treatment. These data demonstrated that hypoxia stimulation induced SDF-1 production in FLSs through activation of NF-κB signaling pathway.

SDF-1/CXCR4 influences a variety of behaviors in cells, including cell migration and metastasis [27–29]. In mechanisms of SDF-1/CXCR4 -mediated metastasis, CXCR4-expressing cells are attracted to a target organ that secretes SDF-1 by sensing a chemokine gradient [27]. It have been reported that SDF-1/CXCR4 was involved in the migration of T cell and monocytes in synovial tissue [30, 31]. In our work, we observed abundant expression of CXCR4 in monocytes. The role of SDF-1/CXCR4 in the migration of monocytes was detected in vitro. The results showed that hypoxia-disposed FLSs could significantly induced the migration of monocytes and the migratory ability of monocytes was abolished when CXCR4 expression was inhibited. These data exhibited that SDF-1 which produced by hypoxia-treated FLSs, attracting CXCR4-expressing monocytes to synovial tissues. Clinically, inhibition of SDF-1/CXCR4 axis would be the effective way to suppress the migration of monocytes in synovial tissues of RA patients.

Once extravagated in the synovial membrane, monocytes were activated and differentiated into tissue macrophages. Macrophages are innate immune cells that play an important role in synovial inflammation and tissue destruction in RA [9]. Macrophages contribute to RA pathogenesis in part by producing key inflammatory cytokines, such as TNF-α, IL-1β and IL-6 [32, 33]. We found that RA-FLSs could induce the differentiation monocytes into macrophages and produce inflammatory factors, such as IL-6, TNF-α, IL-1β and MMP-3, which contributed to the synovial inflammation and tissue destruction in RA. Therefore, strategies that inhibit monocytes migration and differentiation may contribute to the therapeutic efficacy of RA.

## Conclusion

In summary, our data showed that hypoxia microenvironment induced SDF-1 production in FLSs, which lead to the migration of CXCR4-expressed monocytes. Moreover, monocytes were differentiated into tissue macrophage by the interaction with RA-FLSs and secreted inflammatory factors. These data suggested that SDF-1/CXCR4 axis might be a powerful way of inhibiting inflammation and bone erosion in RA.
